# Unveiling the Effects of Two Polycyclic Aromatic Hydrocarbons and Two Temperatures on the Trout RTL-W1 Cell Line Expression of Detoxification-Related Target Genes

**DOI:** 10.3390/jox15030084

**Published:** 2025-06-01

**Authors:** Margarida Vilaça, Telma Esteves, Rosária Seabra, Eduardo Rocha, Célia Lopes

**Affiliations:** 1 Laboratory of Histology and Embryology, Department of Microscopy, ICBAS—School of Medicine and Biomedical Sciences, University of Porto, 4050-313 Porto, Portugal; up201804847@edu.icbas.up.pt (M.V.); up201805154@edu.fc.up.pt (T.E.); rcseabra@icbas.up.pt (R.S.); erocha@icbas.up.pt (E.R.); 2 Team of Animal Morphology and Toxicology, CIIMAR/CIMAR—Interdisciplinary Centre of Marine and Environmental Research, University of Porto, 4450-208 Porto, Portugal

**Keywords:** benzo[k]fluoranthene, benzo[a]pyrene, detoxification, global warming, RTL-W1 liver cell line

## Abstract

Polycyclic aromatic hydrocarbons (PAHs), prevalent aquatic contaminants, arise from burning fossil fuels, a major source of greenhouse gases driving global warming. PAHs and warmer temperatures individually exert diverse negative effects on aquatic organisms. However, the effects of PAH exposure and/or rising temperature remain largely unknown. Liver in vitro models, like the rainbow trout (*Oncorhynchus mykiss*) RTL-W1 liver cell line, have been employed to unravel PAH-exposure effects, primarily on cell viability and enzymatic activity. Here, monolayer-cultured (2D) RTL-W1 cells were used to assess the co-exposure effects of temperature (18 and 21 °C) and two PAHs, benzo[a]pyrene (B[a]P) and benzo[k]fluoranthene (B[k]F), at 10 and 100 nM. After a 72 h exposure, the cell density and viability were evaluated using the trypan blue and LDH assays. The mRNA levels of the detoxification-associated genes *aryl hydrocarbon receptor* (*AhR*), *cytochrome P450 *(*CYP*)*1A*, *CYP3A27*, *glutathione S-transferase omega 1* (*GSTO1*), *uridine diphosphate–glucuronosyltransferase* (*UGT*), *catalase* (*CAT*), and *multidrug resistance-associated protein 2* (*MRP2*) were measured by RT-qPCR. Temperature influenced cell viability and LDH leakage. Both PAHs reduced the cell density and upregulated the mRNA levels of *AhR*, *CYP1A, CYP3A27*, and *UGT*, while *GSTO1* and *MRP2* were only augmented after the higher B[k]F concentration. Temperature influenced *CAT* and *UGT* expression. There was no interaction between temperature and the PAHs. Overall, the results show that B[k]F has more effects on detoxification targets than B[a]P, whereas a temperature increase mildly affects gene expression. The RTL-W1 in 2D seems useful for unravelling not only the liver effects of PAH but also the impact of temperature stress.

## 1. Introduction

Polycyclic aromatic hydrocarbons (PAHs) are widely prevalent in aquatic ecosystems. These hydrophobic compounds, which originate from the pyrolysis of organic matter, are of high concern because they can act as mutagens and carcinogens, including in fish species [1,2]. In vivo, PAHs have also been shown to exert detrimental effects on fish, impacting lipid metabolism, bone remodeling, and the immune and endocrine systems [1].

At the cellular level, PAHs elicit their toxic effects after binding and thus activating the aryl hydrocarbon receptor (AhR), a transcription factor involved in the regulation of the expression of detoxification enzymes, like the well-known phase I cytochrome P450 (CYP) 1A [3]. Phase II enzymes, including uridine diphosphate-glucuronosyltransferases (UGTs) and glutathione S-transferases (GSTs), also metabolize PAHs, making them more polar to facilitate their excretion by efflux transporters [3,4]. The biotransformation reactions, especially those catalyzed by CYP1A, can increase reactive oxygen species (ROS) levels [5], also contributing to PAHs’ toxicity by deregulating the antioxidant enzymes, such as catalase (CAT), superoxide dismutase, and glutathione peroxidase [6,7].

As the prime site where detoxifying reactions occur, the liver has been a central target organ for studies regarding PAHs’ effects on fish, performed both in vivo [8,9,10] and in vitro [5,6]. In fact, various liver in vitro models, including 2D and 3D cultures of primary cells or cell lines [11] and precision-cut liver slices (PCLSs) [12,13], have contributed to unveiling PAHs’ effects at the molecular and cellular levels, specifically characterizing the mechanisms of action.

As an example, the RTL-W1 cell line has been extensively utilized to assess PAHs’ effects, both in single exposures [14,15,16] and in complex mixtures [17]. The cell line, established by Lee et al. (1993) [18] from a non-neoplastic liver of an adult male rainbow trout (*Oncorhynchus mykiss*), expresses AhR [16] and presents highly inducible CYP1A activity [14,15,18]. Indeed, most of the works using cell lines to study PAHs’ effects assess CYP1A activity (measured by EROD assay); mRNA and protein levels [11,14,15,17]; AhR, mainly evaluating its mRNA levels [16]; and PAH-induced oxidative stress [7]. It is, therefore, of utmost importance to evaluate the potential of other PAH targets to be used as exposure biomarkers.

Another threat that aquatic ecosystems cope with is the increase in temperature due to global warming. Indeed, the Intergovernmental Panel on Climate Change [19] report states that the global temperature can reach +4 °C by 2100 (compared to the Pre-Industrial Era global temperature) and that the temperature of aquatic ecosystems will increase accordingly [19]. Temperature changes have been shown to exert effects on fish reproduction [20,21], growth [22,23,24], immune system responses [25], and stress-hormone levels [26]. Fish are ectotherms, so in warmer waters, their corporal temperature increases. At the cellular level, the elevated temperature can accelerate biochemical reaction rates, eventually interfering with xenobiotic metabolism. Despite the known temperature effects, only a few studies using in vitro models studied the harmful effects of increased temperatures on fish [27,28] or the combined effects of high temperatures and contaminants [29,30]. So, additional research is still required to improve the use of in vitro models to study pollutants’ effects in the context of global warming.

In vivo, temperature has been shown to modify PAHs’ effects on fish. As an example, when adult polar cod (*Boreogadus saida*) were exposed to PAH-rich crude oil at 4 °C and 11 °C, the gene expression of *CYP1A*, *UGT*, *GST*, ABC efflux transporters, and some chaperones was significantly upregulated due to the oil uptake, with the higher temperature intensifying the oil’s effects [31]. In line with the previous study, after exposure to PAHs, EROD activity was more strongly induced at 24 °C than at 12 °C in juvenile rainbow trout [32] and at the highest temperatures (7 and 10 °C vs. 2 °C) in juvenile Atlantic cod (*Gadus morhua*) [33]. So, at least in vivo, the combined effects of PAHs and increased temperature seem more detrimental than the isolated effects of each stressor. While more in vivo research is needed to obtain sounder knowledge, the joint effects of both stressors remain even less characterized in vitro.

To address this gap in the literature, this study used 2D-cultured RTL-W1 cells to explore the effects of PAHs, namely benzo[k]fluoranthene (B[k]F) and benzo[a]pyrene (B[a]P), and temperature (18 °C vs. 21 °C) on the cell density, viability, and mRNA expression of a set of detoxification genes. To our best knowledge, this is a pioneer study unveiling these mixed effects using an in vitro 2D model, making the data obtained here a reference point for further studies on the independent and combined effects of PAHs and increased temperatures in vitro.

## 2. Materials and Methods

### 2.1. The Culture of the RTL-W1 Cell Line

The RTL-W1 cell line (Cellosaurus, www.cellosaurus.org/index.html (accessed on 14 March 2025), CVCL_L011) was a gift from Dr. Lucy E. Lee and Niels C. Bols. The RTL-W1 cells were grown in T75 culture flasks (5520200, Orange Scientific, Braine-l’Alleud, Belgium) with 15 mL of phenol red-free Leibovitz’s (L-15) culture medium (21083-027, Gibco^TM^, Thermo Fisher Scientific, Grand Island, NY, USA), supplemented with 5% (*v*/*v*) fetal bovine serum (FBS) (F9665, Sigma-Aldrich, St. Louis, MO, USA) and 1% (*v*/*v*) of a stock solution of 10,000 U/10,000 µg/mL penicillin/streptomycin (A2213, Merck KGaA, Darmstadt, Germany). The cells were maintained at 18 °C in a cooled incubator (Heraeus, BK 6160, Thermo Scientific, Langenselbold, Germany) without a CO_2_ supply, with a medium change every two to three days. When needed, subculturing using 0.05/0.02% trypsin/ethylenediaminetetraacetic acid (EDTA) (59418C, Sigma-Aldrich, St. Louis, MO, USA) was performed.

### 2.2. Exposure to PAHs

To study whether temperature influences the effects of B[a]P and B[k]F on RTL-W1 cells, we conducted 5 independent experiments (performed on different days) per temperature. For each experiment, three 6-well plates (4430500, Orange Scientific, Braine-l’Alleud, Belgium) were used. Per well, 4.8 × 10^5^ cells—whose passages ranged from 110 to 120—were seeded in 3 mL of supplemented L-15 medium. The cells were then incubated at 18 °C (Heraeus BK 6160, Thermo Scientific, Langenselbold, Germany) or 21 °C (Incu-Line^®^ 150R Premium, VWR International, Leuven, Belgium), depending on the experiment.

The B[a]P (CAS 50-32-8, Sigma-Aldrich, St. Louis, MO, USA) and B[k]F (CAS 207-08-9, Sigma-Aldrich, St. Louis, MO, USA) exposure solutions were prepared from 0.001 M stock solutions in dimethylsulfoxide (DMSO) (0231-500ML, VWR, Solon, OH, USA). All three plates in each experiment (technical replicates) encompassed the 6 exposure conditions: control (C)—complete L-15 medium; solvent control (SC)—0.1% DMSO in complete L-15 medium; 10 nM of B[a]P (B[a]P10); 100 nM of B[a]P (B[a]P100); 10 nM of B[k]F (B[k]F10); and 100 nM of B[k]F (B[k]F100). The exposure media (all but the C) were diluted from the 0.001 M stocks in the complete L-15 medium, assuring a DMSO concentration of 0.1%. The exposures started after a 24 h adherence period and lasted 72 h, with daily renewals of the exposure media. The plate design was randomized, i.e., the different exposure conditions were designated arbitrarily to the plate wells. The tested concentrations were selected to cover the reported ranges of EROD induction by B[a]P and B[k]F in primary rainbow trout hepatocytes and in RTL-W1 cells [14,15].

After 72 h of exposure, media from the different conditions were collected for the assessment of lactate dehydrogenase (LDH) release. The cells were then trypsinized and counted, and cell viability and cell density were assessed in the cell suspensions, in duplicate per condition/plate, using the trypan blue exclusion assay [34].

After the cell viability assessment, the cells were pelleted by centrifuging at 200× *g*, 5 min at 15 °C (521-1895 Mega Star, VWR International, Leuven, Belgium), giving a total of 3 pellets per condition, one from each plate, for the gene expression analyses; these were frozen in liquid nitrogen (after removing the supernatant) and kept at −80 °C until RNA extraction.

### 2.3. Lactate Dehydrogenase (LDH) Assay

The LDH assay (ENZ-KIT157-2500, Enzo Life Sciences, Farmingdale, NY, USA) was used to evaluate cell viability in triplicate per condition/plate. The cell culture media from each PAH exposure condition (600 µL) were collected into non-sterile microtubes and centrifuged (200 × g, 5 min; MicroStar 12, VWR International, Leuven, Belgium), and then 100 µL of medium from each condition (× 3) was pipetted to a 96-well plate (4430100, Orange Scientific, Braine-l’Alleud, Belgium). The working solutions of each condition were used for background controls (also in triplicate). Afterwards, 100 µL of assay buffer was pipetted to each well, and the plate was incubated in the dark for 30 min at room temperature. Finally, 50 µL of stop solution was added to each well, and the absorbance was measured, at 490 nm, in the Multiskan GO spectrophotometer (Thermo Fisher Scientific Oy, Vantaa, Finland). The absorbances of each condition were corrected by subtracting the respective background control and plotted for the different exposure conditions, as described elsewhere [35,36].

### 2.4. Molecular Analysis

#### 2.4.1. RNA Extraction and cDNA Synthesis

The RNA was extracted from the cell pellets using the illustra^TM^ RNAspin Mini Isolation Kit (25-0500-72, GE Healthcare, Amersham, UK). The RNA integrity was confirmed by running samples in a 1% agarose gel using GelRed as a fluorescent nucleic acid stain (Biotium, Fremont, CA, USA). Quantification was performed in the Multiskan Go spectrophotometer (Thermo Fisher Scientific Oy, Vantaa, Finland) by loading samples into the μDrop™ Plate (Thermo Fisher Scientific, Singapore). The purity of the nucleic acid samples was assessed by analyzing the 260/280 and 260/230 absorbance wavelength ratios. As the mRNA quality parameters were equivalent between the samples, cDNA was synthesized from 500 ng of total RNA, taken from two replicates per condition per experiment, using the iScript^TM^ Reverse Transcription Supermix for RT-qPCR (1708841, Bio-Rad, Hercules, CA, USA).

#### 2.4.2. Reverse Transcription–Quantitative Polymerase Chain Reaction (RT-qPCR)

The gene expression was analyzed by using the thermal cycler CFX Connect™ (Bio-Rad, Hercules, CA, USA). Before the RT-qPCR analysis, calibration curves were performed to calculate the reaction efficiencies for each primer pair. The protocol details for the selected genes are listed in Table 1.

The samples were loaded in duplicates into the RT-qPCR plates, which also included no-template controls (nuclease-free water instead of cDNA) and a calibrator consisting of cDNA from 4 random samples. The 20 μL reaction mixtures encompassed 10 μL of iQ™ SYBR^®^Green Supermix (1708886, Bio-Rad, Hercules, CA, USA), 200 nM of gene-specific primers (Table 1), and 5 μL of cDNA diluted 1:10 in nuclease-free water. The melt curve generated at the end of each amplification cycle allowed us to assess that a single amplicon was being amplified.

The gene expression of the target genes was normalized using the geometric mean of the reference genes *ef1α* and *β-actin*, as their expression was stable between temperatures and PAH exposure conditions. Relative quantification was performed by the Pfaffl method [37].

**Table 1:** Target genes, primer sequences, amplification efficiencies (E), and detailed protocols used in the RT-qPCR. Annealing temperatures are shown in bold.

Gene	Primer (5′-3′)	Protocol	E (%)	Reference
* AhR *	F: GGATGCCACTGAGTTCCAAACCAA R: AATGCCTGGTCTATGGGTAGCTGA	95 °C—3 min (95 °C—20 s; **60.0 °C**—20 s; 72 °C—20 s) 40× 95 °C—1 min	101.6	[38]
* CYP1A *	F: GATGTCAGTGGCAGCTTTGA R: TCCTGGTCATCATGGCTGTA	95 °C—3 min (95 °C—20 s; **60.0 °C**—20 s; 72 °C—20 s) 40× 95 °C—1 min	97.7	[39]
* CYP3A27 *	F: GACGGTGGAGATCAACG R: GAGGATCTCGACCATGG	95 °C—3 min (95 °C—20 s; **60.0 °C**—20 s; 72 °C—20 s) 40× 95 °C—1 min	99.0	[39]
* UGT *	F: ATAAGGACCGTCCCATCGAG R: ATCCAGTTGAGGTCGTGAGC	94 °C—3 min (94 °C—20 s; **60.0 °C**—20 s; 72 °C—20 s) 40× 94°C—1 min	94.9	[39]
* GSTO1 *	F: AGCTGCTCCCAGCTGATCC R: CAAACCACGGCCACATCATGTAATC	94°C—3 min (94 °C—20 s; **60.0 °C**—20 s; 72 °C—20 s) 40× 94 °C—1 min	93.9	[38]
* CAT *	F: CACTGATGAGGGCAACTGGG R: CTTGAAGTGGAACTTGCAG	95 °C—3 min (95 °C—10 s; **58.0 °C**—30 s; 72 °C—30 s) 40× 95 °C—30 s	104.5	[40]
* MRP2 *	F: CCATTCTGTTCGCTGTCTCA R: CTCGTAGCAGGGTCTGGAAG	94 °C—3 min (94 °C—20 s; **60.0 °C**—20 s; 72 °C—20 s) 40× 94 °C—1 min	100.2	[39]
* β-act *	F: TCTGGCATCACACCTTCTAC R: TTCTCCCTGTTGGCTTTGG	94 °C—3 min (94 °C—20 s; **55.0 °C**—20 s; 72 °C—20 s) 40× 94 °C—1 min	99.7	[41]
* ef1α *	F: TGCCACACTGCTCACATC R: TCTCCAGACTTCAGGAACTTG	94 °C—3 min (94 °C—20 s; **55.0 °C**—20 s; 72 °C—20 s) 40× 94 °C—1 min	97.4	[42]

*AhR* (aryl hydrocarbon receptor), *CYP1A* (cytochrome P450 1A), *CYP3A27* (cytochrome P450 3A27), *UGT* (uridine diphosphate (UDP)-glucuronosyltransferase), *GSTO1* (glutathione S-transferase omega 1), *CAT* (catalase), *MRP2* (multidrug resistance-associated protein 2), *β-act* (beta-actin), *ef1α* (Elongation Factor 1 alpha).

### 2.5. Statistical Analysis

The Jamovi software (version 2.3) was used for the statistical analysis. Data sets were tested for normality and the homogeneity of variances using the Shapiro–Wilk W and Levene’s tests, respectively. The data were then transformed as needed to meet normality and variance homogeneity requirements. Subsequent two-way ANOVA analyses were followed by Tukey’s pairwise comparisons. As there were no significant differences between the data from the C and SC conditions, they were treated as one group (C condition) in the statistical analysis [43]. Differences were considered significant whenever *p* < 0.05. The computer program GraphPad Prism (Version 9.0) was used to prepare the presented graphs.

## 3. Results

### 3.1. Cell Density and Viability

**Figure 1:**
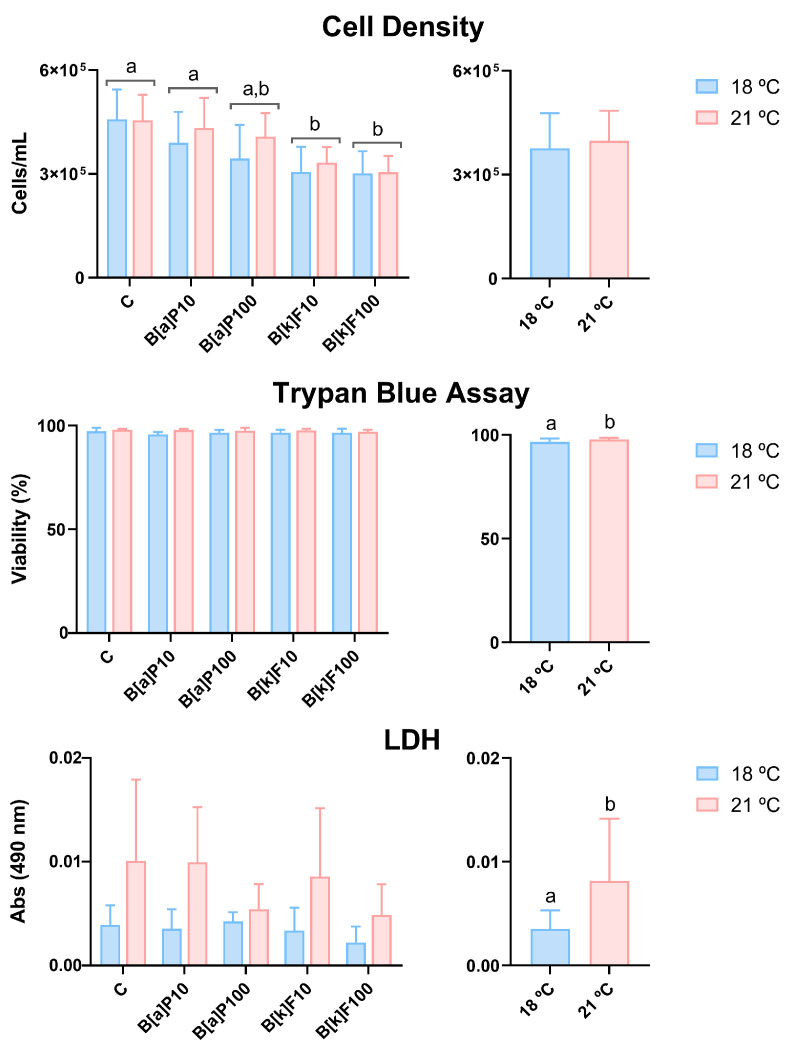
Cell density and viability (evaluated by trypan blue exclusion and LDH assays) after 72 h of exposure to benzo[a]pyrene (B[a]P) or benzo[k]fluoranthene (B[k]F) at 18 °C or 21 °C. C—control; B[a]P10—10 nM of B[a]P; B[a]P100—100 nM of B[a]P; B[k]F10—10 nM of B[k]F; B[k]F100—100 nM of B[k]F. The data are presented as mean ± standard deviation. The graphs on the left present the data plotted against the different exposure conditions, while the graphs on the right present the data grouped by temperatures. Conditions or temperatures marked with different lowercase letters are significantly different (e.g., a vs. b), whereas those sharing a common letter (e.g., a vs. a,b) do not differ statistically, according to a two-way ANOVA followed by Tukey’s test. N = 5 independent experiments per temperature.

Cell density significantly decreased with B[k]F exposure, as the control differed significantly from the B[k]F10 and B[k]F100 groups (Figure 1). However, it was not significantly affected by B[a]P, although a decreasing trend was also noted, or temperature. Concerning cell viability, globally, it was statistically higher at 21 °C than at 18 °C, but it was not significantly affected by the exposure to either PAH (Figure 1). In the LDH assay, the LDH leakage was low, as can be inferred by the measured absorbance values (Figure 1). Overall, LDH levels were significantly increased at 21 °C compared with 18 °C but unaffected by the exposures to either PAH.

### 3.2. Gene Expression Analysis

**Figure 2:**
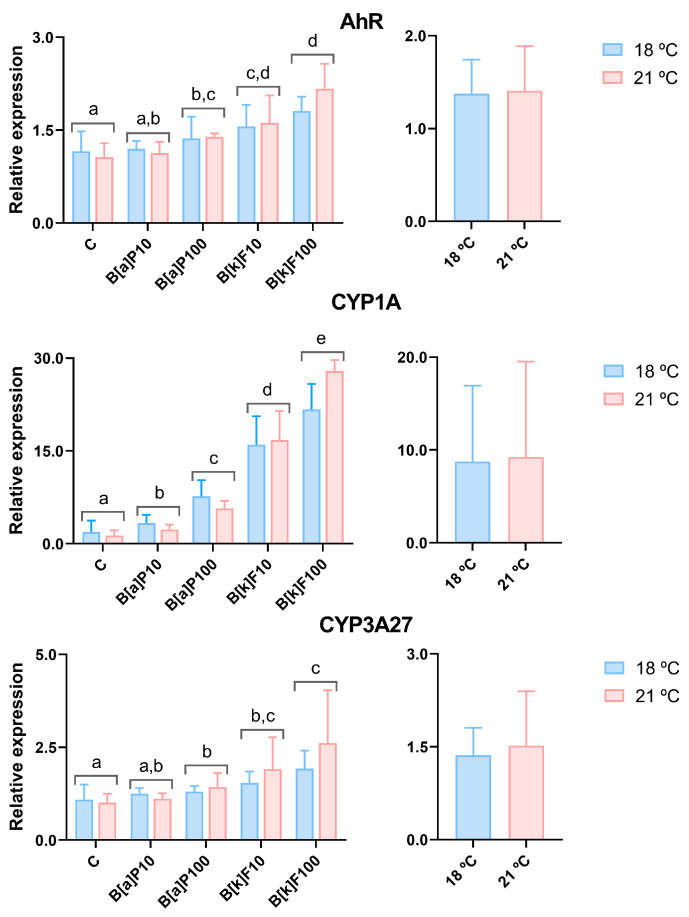
The relative gene expression of detoxification target genes in RTL-W1 cells after 72 h of exposure to benzo[a]pyrene (B[a]P) or benzo[k]fluoranthene (B[k]F) at 18 °C or 21 °C. C—control; B[a]P10—10 nM of B[a]P; B[a]P100—100 nM of B[a]P; B[k]F10—10 nM of B[k]F; B[k]F100—100 nM of B[k]F. The data concerning the *AhR* (*aryl hydrocarbon receptor*), *CYP1A* (*cytochrome P450 1A*), and *CYP3A27* (*cytochrome P450 3A27*) relative expression levels are presented as the mean ± standard deviation. The graphs on the left present the data plotted against the different exposure conditions, while the graphs on the right present the data grouped by temperatures. Conditions marked with different lowercase letters are significantly different (e.g., a vs. b), whereas those sharing a common letter (e.g., a vs. a,b) do not differ statistically, according to a two-way ANOVA followed by Tukey’s test. N = 5 independent experiments per temperature.

**Figure 3:**
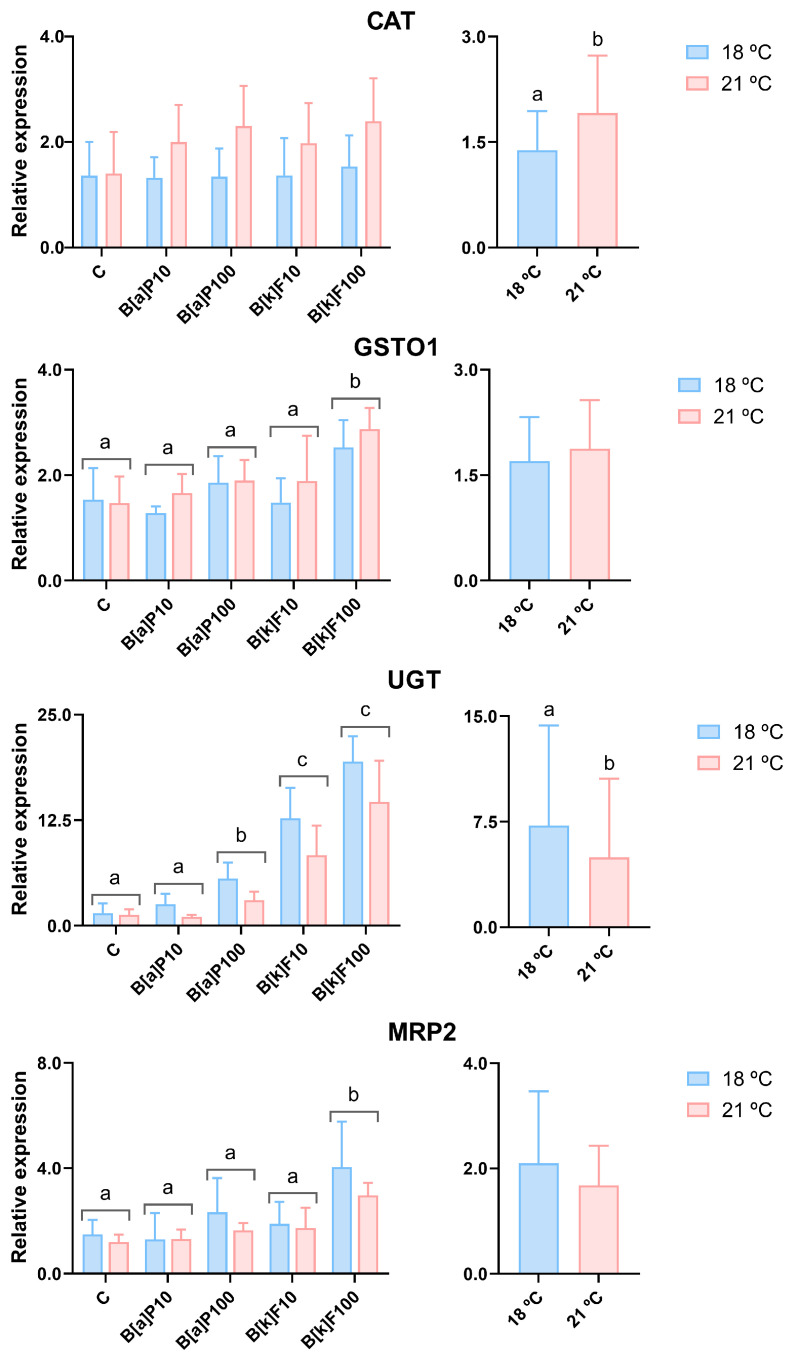
The relative gene expression of detoxification target genes in RTL-W1 cells after 72 h of exposure to benzo[a]pyrene (B[a]P) or benzo[k]fluoranthene (B[k]F) at 18 °C or 21 °C. C—control; B[a]P10—10 nM of B[a]P; B[a]P100—100 nM of B[a]P; B[k]F10—10 nM of B[k]F; B[k]F100—100 nM of B[k]F. The data concerning the *CAT* (*catalase*), *GSTO1* (*glutathione S-transferase omega 1*), *UGT* (*uridine diphosphate (UDP)-glucuronosyltransferase*), and *MRP2* (*multidrug resistance-associated protein 2*) relative expression levels are presented as the mean ± standard deviation. The graphs on the left present the data plotted against the different exposure conditions, while the graphs on the right present the data grouped by temperatures. Conditions or temperatures marked with different lowercase letters are significantly different (e.g., a vs. b), according to a two-way ANOVA followed by Tukey’s test. N = 5 independent experiments per temperature.

The relative gene expression of the RTL-W1 cells exposed to B[a]P or B[k]F at 18 °C or 21 °C is represented in Figure 2 and Figure 3. The mRNA levels of *AhR* were increased by B[a]P100 and by both concentrations of B[k]F compared with the C (Figure 2). In relation to *CYP1A*, all the exposed groups differed from the C. Additionally, the highest concentration of both compounds induced higher mRNA levels of this gene than the lowest one, denoting a dose-dependent effect (Figure 2). Moreover, the lowest concentration of B[k]F induced higher levels of *CYP1A* than either of the B[a]P concentrations. *CYP3A27* mRNA levels significantly increased at the highest B[a]P concentration and the two B[k]F concentrations compared to the C (Figure 2). Neither of the *CYPs*’ expression was significantly impacted by temperature, and no combined effects of temperature and the tested PAHs were verified.

The exposure to B[a]P or B[k]F did not influence the mRNA levels of *CAT*, but its relative expression was impacted by temperature, being overall significantly higher at 21 °C than at 18 °C (Figure 3). *GSTO1* mRNA levels were only increased above the C levels by B[k]F100 (Figure 3). However, exposure to either of the PAHs and temperatures influenced the expression of the other phase II biotransformation enzyme, *UGT*. Specifically, the highest concentration of B[a]P and the two concentrations of B[k]F induced the expression of *UGT,* and the expression at 18 °C was higher than that at 21 °C (Figure 3). As for *MRP2*, the factor temperature did not influence its relative expression, but B[k]F100 significantly increased its expression compared to all the other conditions (Figure 3).

## 4. Discussion

### 4.1. Cell Viability Tests

The cell density was decreased in both concentrations of B[k]F, whereas the viability percentages did not differ between exposure conditions, as measured by the trypan blue exclusion assay. These results may seem contradictory, as the decrease in cell density suggests that at least B[k]F exposure increased the cell mortality. Although not significant, a decreasing trend in cell density was also noted in the higher B[a]P concentration, which was also noted in the microscopic observation of the culture plates. When cultured in a monolayer, dead cells detach from the bottom of the flask or plate wells, so here they were probably removed with the medium changes (every 24 h) and washing steps before trypsinization at the end of some of the exposures. The aspiration of the dead cells may thus explain the lower densities in the B[a]P100- and B[k]F-exposed conditions. In PLHC-1 cells from topminnow fish (*Poeciliopsis lucida*) exposed in a monolayer to either 1 µM or 5 µM B[a]P, a decrease in the cell number was found after a 24 h exposure [11]. Also, B[a]P (39.6 pM–39.6 µM) induced apoptosis in monolayer-cultured primary hepatocytes from gilthead sea bream (*Sparus aurata*), also after a 24 h exposure [44]. So, it can be argued that higher doses of the tested compounds could decrease the cell density even more.

The cell viability measured by the trypan blue assay was consistently excellent (> 90%) and even increased at 21 °C. Similarly, LDH levels were higher at 21 °C than at 18 °C. However, an increase in LDH levels is usually indicative of decreased cell viability. Even though there were no significant statistical differences, the descriptive statistics suggested a slightly higher number of cells at 21 °C than at 18 °C. If more cells release LDH, the increase in absorbance can be explained by the slightly higher number of cells in this condition. The latter is a likely explanation, given that we are dealing with generally very low LDH levels. In fact, these very low absorbance levels support no membrane damage and, therefore, align with the high viability of the cells in all the conditions in this experiment.

Corroborating this result, 3D-cultured RTL-W1 cells exposed to 10 or 100 nM of B[k]F (4-day exposure) at 18 or 23 °C also presented very low LDH levels, which did not differ between the B[k]F exposure conditions [35].

It might be argued that although viability was consistently high in the employed viability tests, the cells’ metabolic activity could even so be compromised by the PAH exposure. Although this endpoint was not assessed in the present study, RTL-W1 spheroids exposed to the same B[k]F concentrations employed here showed no differences in viability with the alamarBlue assay, which infers mitochondrial metabolic activity [35]. The same was verified in 2D-cultured RTL-W1 cells, which showed viabilities higher than 90% in a similar viability assay (also based in resazurin reduction) after exposure to up to 1 µM of B[a]P [45].

### 4.2. Gene Expression 

The RTL-W1 cell line was overall sensitive to the tested PAHs, with changes in the mRNA levels of all the detoxification target genes except *CAT*.

In relation to *CAT* expression, it seems that the used concentrations of B[a]P and B[k]F could not increase ROS production, namely hydrogen peroxide, which is the main substrate of CAT, at least to the point of upregulating the gene. CAT was not considered a reliable biomarker of exposure to PAHs since it presented a remarkable variability between different in vivo studies [46]. However, even though the general pattern for *CAT* here lacks significant responses after PAH exposure, the effects of the PAHs on *CAT* can depend on the chosen model. For example, in primary hepatocytes of orange-spotted grouper (*Epinephelus coioides*), CAT activity increased after exposure to 10, 20, and 30 µM of B[a]P for 6, 12, and 24 h [6]. Additionally, in vivo, increases of *CAT* expression were observed in polar cod exposed (16 h—4 d) to B[a]P (6.6, 85 and 378 µg/kg wet weight) [47]. So, it cannot be ruled out that a higher concentration of B[a]P or B[k]F or even measuring the gene expression at a different time point could lead to significant differences in *CAT* expression in our model. Regarding temperature effects, *CAT* mRNA levels were more upregulated at 21 °C compared to 18 °C. This result aligns with findings from some in vivo studies on the impacts of increased temperatures on liver CAT activity and mRNA levels. Specifically, increased CAT activity at 21 °C versus the 15 °C control was observed in Atlantic salmon (*Salmo salar*) after 43 days of exposure [48], while rainbow trout exhibited elevated *CAT* mRNA levels after 22 °C exposure for 30 days in comparison with the 17 °C control [49].

All the concentrations of both compounds increased *CYP1A* expression in a concentration-dependent manner, reinforcing the use of this gene as the biomarker of excellence of PAH exposure in RTL-W1 cells. Also, the lowest B[k]F concentration induced higher levels of *CYP1A* than either of the B[a]P concentrations. This result is in line with the finding that B[k]F was a more potent inducer of RTL-W1 cells’ EROD activity than B[a]P [15]. Unlike *CYP1A*, the *AhR* expression was not induced by the lowest B[a]P concentration. Also, the fold-change of *AhR* expression was almost 10 times lower than *CYP1A*’s. Similarly, in polar cod exposed intraperitoneally to B[a]P (6.6, 85, and 378 µg/kg wet weight), *AhR* expression remained unchanged, while *CYP1A*’s was significantly upregulated by the two highest concentrations [47]. Neither CYP1A nor AhR genes were impacted by temperature, and synergistic effects were not detected. On the contrary, a higher temperature intensified PAH-induced EROD activity in juvenile rainbow trout—24 vs. 12 °C [32]—and in juvenile Atlantic cod—10 °C vs. 2 and 7 °C [33]—as well as oil-induced *CYP1A* expression in adult polar cod—11 vs. 4 °C [31]—and embryos—2.8 vs. 0.5 °C [50]. At least in vivo, it seems that temperature modulates hepatic biotransformation processes in various fish species. Still, we could not replicate the same kind of effect with the employed in vitro model and exposure conditions.

Regarding *CYP3A27*, both concentrations of B[k]F and 100 µM B[a]P increased its expression. Enzymes belonging to the CYP3A family are mainly activated through the pregnane X receptor (PXR) pathway [38] but can also be activated by the AhR pathway [51,52]. In line with our results, 0.3 nM–1 µM of B[k]F, B[a]P, benzo[a]anthracene, and dibenzo[a]anthracene induced the activity of 7-benzyloxy-4-trifluoromethylcoumarin-*O*-debenzylation (BFCOD), used as a proxy of CYP3A activity, in RTL-W1 cells, after 4, 24, and 48 h of exposure [51]. In the present work, *CYP3A27* levels after PAH exposure were much lower than *CYP1A’s*, suggesting that even though *CYP3A27* is an important phase I enzyme, it may not be a first-choice biomarker of PAH exposure.

Even though GST activity was considered a reliable biomarker of exposure to PAHs [46], in this study, only B[k]F100 induced the expression of *GSTO1*. *UGT,* the other phase II enzyme, was more sensitive to the PAHs than *GSTO1*, which is similar to what was found in RTL-W1 spheroids after exposure to B[k]F [35]. Fish—and also RTL-W1 cells—express phenol-type UGTs, responsible for the glucuronidation of B[a]P and other PAHs [53,54], which is considered an important step in the metabolization of these compounds [55]. So, the upregulation of *UGT* in the RTL-W1 cells may be related to an intent to metabolize the PAHs better, facilitating their further excretion. In addition, *UGT* expression was influenced by temperature, with a higher expression at 18 °C than 21 °C but without interactive effects between temperature and the PAHs. On the contrary, when Gulf killifish (*Fundulus grandis*) larvae were exposed to crude oil (rich in PAHs) at two temperatures (20 and 30 °C), *UGT1A1* upregulation due to oil exposure was intensified at the highest temperature [56]. Our result suggests the PAHs’ glucuronidation by UGT may be disturbed at the highest temperature, eventually contributing to lower cellular metabolite export rates at that temperature in this model. Notably, in the same model exposed to ethynylestradiol and levonorgestrel, an increasing trend (close to significancy) towards increased *UGT* levels at 21 °C was found [30]. So, it can be inferred that various chemicals may influence the temperature effects on UGT expression. Accordingly, it would be interesting for future studies to examine these interactions more deeply.

High *MRP2* expression was previously reported in the RTL-W1 cell line [4]. Given the MRP2 role in the transport of various substrates across cellular membranes, it may be theorized in the present study that the greater expression of *MRP2* in the higher B[k]F concentration may serve an enhanced cell need to excrete B[k]F metabolites at this concentration. Curiously, the expression pattern of *MRP2* aligns with the pattern of *GSTO1*, indicating that the glucuronidation of the B[k]F by GST can facilitate B[k]F excretion by MRP2. Nevertheless, temperature did not exert effects on *MRP2*’s nor *GSTO1*’s expression, suggesting that, at least at the time point measured, the increased temperature did not accelerate B[k]F excretion. In adult polar cod exposed to crude oil at 4 and 11 °C, the oil-induced liver expression of some ABC efflux pumps, including MRP2, was intensified at 11 °C compared to 4 °C [31]. In RTL-W1 spheroids, the expression of *MRP2* was influenced by temperature, decreasing at 23 °C compared to 18 °C, but was not influenced by B[k]F exposure [35]. So, further studies using different models and species are needed to explore PAHs’ influences on efflux transporters’ expression or activity and possible temperature interactions.

The heat shock employed in this work is environmentally relevant since it scopes in the worst-case scenario predictions for climate change [19]. However, it is small compared to other studies that, using a wider range, reported temperature effects on some targets studied here. So, despite being realistic, the 3 °C temperature difference used here was probably insufficient to obtain more detectable effects. The overall reduced temperature impacts in gene expression and the lack of interaction effects can also be partially explained by the fact that, at least for other gene targets (namely heat-shock proteins), increased temperatures may exert their effects earlier than the timepoint measured here [57,58].

## 5. Conclusions

When the RTL-W1 cell line was exposed to B[a]P and B[k]F at the two temperatures, the PAHs reduced the cell density, while the higher temperature increased the cell viability (as assessed by the trypan blue exclusion assay). Also, PAH-induced changes were observed in the expression (mRNA levels) of all the detoxification target genes except *CAT*. Temperature had milder effects on gene expression, altering the expression of *CAT* and of the phase II detoxification enzyme *UGT*. So, this study reinforced this cell line’s usefulness in studying PAH-induced alterations in detoxification pathways. The temperature effects on gene expression have the potential to be explored, especially using a wider range of temperatures and different exposure times. Future studies could also explore changes at the protein level.

## Data Availability

The original contributions presented in this study are included in the article. Further inquiries can be directed to the corresponding author(s).
